# PHF5A facilitates the development and progression of gastric cancer through SKP2-mediated stabilization of FOS

**DOI:** 10.1186/s12967-022-03821-w

**Published:** 2023-01-06

**Authors:** Zhandong Zhang, Liangqun Peng, Wei Yang, Baodong Li, Yawei Hua, Suxia Luo

**Affiliations:** Department of General Surgery, Henan Tumor Hospital, Affiliated Tumor Hospital of Zhengzhou University, 127 Dongming Road, Zhengzhou, Henan China

**Keywords:** Gastric cancer, PHF5A, FOS, Phenotype, Ubiquitination

## Abstract

**Background:**

Gastric cancer (GC) is the fifth most common cancer and the third most common cause of cancer death worldwide. Plant homeodomain (PHD)-finger domain protein PHF5A has been demonstrated to play a promoting role in a variety of cancers. This study aimed to clarify the role of PHF5A in the progression of GC and its potential mechanism of action.

**Methods:**

Immunohistochemical staining experiments were performed based on tissues from clinical GC patients to reveal PHF5A expression. A series of functional experiments in vitro and in vivo were used to clarify the role of PHF5A in GC.

**Results:**

Clinically, PHF5A was abundantly expressed in GC and existed clinical value indicating poor prognosis. In addition, GC cells with knockdown of PHF5A expression showed slowed proliferation, enhanced sensitivity to apoptosis and inhibition of migration. Mechanically, knockdown of PHF5A led to decreased protein stability of FOS, which was mediated ubiquitination of E3 ubiquitin ligase S-phase kinase-associated protein 2 (SKP2). Moreover, downregulation of FOS attenuated the promotion of PHF5A overexpression on GC cells. Consistently, Pladienolide B (PHF5A inhibitor) treatment reversed the induction of PHF5A overexpression on the malignant phenotypes and tumor formation of GC cells.

**Conclusion:**

Knockdown of PHF5A inhibited the progression of GC through SKP2-mediated ubiquitination of FOS, which may be a promising candidate target with potential therapeutic value.

**Supplementary Information:**

The online version contains supplementary material available at 10.1186/s12967-022-03821-w.

## Introduction

Gastric cancer (GC) is the fifth most common cancer and the third most common cause of cancer death worldwide[1]. The incidence and mortality of this cancer have steadily declined over the past century [2]. Nevertheless, the phenomenon of ageing populations will lead to more cases of GC in the future [3]. It is a molecularly and phenotypically highly heterogeneous disease, which results from the stepwise accumulation of a large number of genetic alterations [4]. The first line of treatment for early GC is endoscopic resection [5]. Advanced GC is treated with continuous chemotherapy, starting from the first line with platinum and fluoropyrimidine [6]. Despite advances in the technology of surgical, radiotherapy, chemotherapy, and neoadjuvant therapy, the prognosis for GC is poor. Recently, chemotherapy combined with molecular-targeted therapy offers survival benefits for patients with advanced GC. Targeted therapies approved for the treatment of GC include epidermal growth factor receptor (EGFR), human epidermal growth factor receptor 2 (HER2) (Trastuzumab), vascular endothelial growth factor (VEGF) (Ramosumab), mammalian target of rapamycin (mTOR) and Claudin 18.2 (CLDN 18.2) [7–9]. However, long-term application of these molecularly targeted agents leads to the emergence of drug resistance in GC patients [10]. Therefore, we need a deep understanding of GC to lay a theoretical foundation for the development of novel molecular-targeted drugs.

Cancer cells often take advantage of the flexibility of aberrant splicing (AS) to produce proteins that promote tumor growth and survival [11, 12]. Plant homeodomain (PHD)–finger domain protein (PHF5A) is involved in AS, which is crucial for the stability of splicing factor 3b (SF3B) and can connect the spliceosome to histones [13]. In addition, PHF5A regulates DNA repair in class switch recombination via p400 and histone H2A variant deposition[14]. This highly conserved protein PHF5A is widely expressed in the nucleus and regulates the pluripotency and cell differentiation phenotype of embryonic stem cells through transcription [15, 16]. PHF5A regulates the viability of glioma pleomorphic stem cells through the cell cycle [17]. Subsequent studies have shown that PHF5A is a key splicing factor involved in tumor progression [18]. Similarly, Mao et al. validated that PHF5A played an oncogenic role in lung adenocarcinoma[19]. Moreover, reduced expression of PHF5A can inhibit the migration and invasion of HCC cells by downregulating NF-B signaling pathway [20]. Wang et al., reported that acetylation of PHF5A modulates stress responses and colorectal carcinogenesis through AS-mediated upregulation of lysine demethylase 3 A (KDM3A) [21]. However, the role of PHF5A in the progression of GC remains unclear.

Here, the clinical value, biological function and underlying mechanism of PHF5A in GC were revealed. Moreover, we established the knockdown of PHF5A in AGS and MGC-803 cells through lentiviral interference, which was used to explore the role of PHF5A in GC. Therefore, the effect of PHF5A in GC progression were preliminarily demonstrated in this study, which may provide a possible target for effective treatment of GC.

## Materials and methods

### Ethics approval

The clinical research protocol was approved by the Institutional Ethics Committee of the Henan Tumor Hospital on March 2, 2021, and informed consent was obtained from all patients (n = 114).

### Tissue samples collection

Tumor tissues (n = 114) and their adjacent normal tissues (n = 112) of GC patients were collected from the Henan Tumor Hospital. The inclusion criteria of the samples were the survival period of GC patients and none of them received any local or systemic treatment before surgery.

### Immunohistochemical staining

The tissue samples were fixed in 4% paraformaldehyde, hydrated through ethanol solution, embedded in paraffin and incubated with 3% hydrogen peroxide. Afterwards, the tissues samples were incubated with the primary anti-PHF5A (1:100 dilution, Abcam, ab193115) anti-FOS (1: 1:500 dilution, Abcam, ab184938), and anti-Ki67 (1:200 dilution, Abcam, ab16667) overnight at 4 °C. After incubation with secondary antibody HRP goat anti-rabbit IgG (1:200 dilution, Beyotime, A0208) at room temperature for 1 h, DAB and hematoxylin was stained. Scoring the intensity of tissue staining based on the criteria provided in the reference [22]. A score greater than or equal to the median of immunohistochemistry indicated high expression of PHF5A, otherwise low expression.

### Cell culture

The human normal gastric mucosa cells GES1 and GC cell lines MGC-803, AGS, SGC-7901, BGC-823 were purchased from Chinese Academy of Sciences (Shanghai, China). All of the cell lines were maintained the Dulbecco’s modified eagle medium supplemented with 10% fetal bovine serum (Invitrogen Gibco) and incubated at 37 °C in a humidified environment with 5% CO_2_.

### RNA interference and cell transfection

According to the gene sequence, small, interfering, specifically targeting human PHF5A (shPHF5A), FOS (shFOS), amplified sequence of PHF5A (PHF5A), and non-specific negative control (shCtrl)were synthesized (Additional file 1: Table S1). AGS and MGC-803 cells were incubated for 24 h and transfected with lentivirus shPHF5A, shFOS and PHF5A (1 × 108 TU/mL) using Lipofectamine® 2000 (Invitrogen; Thermo Fisher Scientific, USA) at a MOI (multiplicity of infection) of 10. Notably, GC cells transfected with lentivirus PHF5A and NC-shFOS for overexpressing PHF5A [PHF5A + NC(KD)]; the cells transfected with lentivirus shFOS and NC-PHF5A for downregulating FOS [shFOS + NC(OE)]; the cells transfected with shFOS and PHF5A for simultaneously downregulating FOS and overexpressing PHF5A [shFOS + PHF5A]; NC(OE + KD) was the cells transfected with empty plasmid, as negative control.

### RNA extraction and qRT-PCR

Total RNA was purified from cultured GC cell lines using Trizol reagent (Invitrogen, Carlsbad, CA, USA). The concentration and purity of RNA samples was assessed with a Nanodrop 2000 spectrophotometer (Thermo Fisher Scientific, USA). RNA was reverse transcribed into cDNA by Maxima First Strand cDNA Synthesis Kit (Thermo Fisher Scientific). We conducted quantitative real-time polymerase chain reaction (qRT-PCR) by using the SYBR Green master mix (Thermo Fisher Scientific). Expression of PHF5A and FOS was assessed by threshold cycle (CT) values and analyzed using the 2^ΔΔCt^ method. Primer sequences were designed (Additional file 2: Table S2) and synthesized by Sheng gong (Guangzhou, China).

### Western blot analysis

Total proteins were purified using RIPA lysis buffer (Beyotime, Jiangsu, China) and the concentrations were detected by protein assay kit (Beyotime, Jiangsu, China). Protein lysates were subjected to 10% SDS-PAGE, transferred to PVDF membrane (Millipore), hybridized with corresponding primary antibody (Additional file 3: Table S3) overnight at 4 °C. The next day, the membranes were incubated with secondary antibody HRP goat anti-rabbit IgG (Beyotime, A0208) at room temperature for 2 h. The coloration of the membrane was performed with chemiluminescence ECL kit (Thermo Fisher Scientific) and protein signal was visualized by Odyssey Infrared scanning system (Li-Cor, Lincoln, NE, USA).

### Co-immunoprecipitation (Co-IP) assay

A target protein-specific anti-PHF5A or anti-FOS antibody (Additional file 3: Table S3) in conjunction with Protein A/G affinity beads (Santa Cruz Biotechnology) for 30–60 min at 4 °C. The bead-antibody complexes were suspended with protein lysate. The beads were washed 3 times with extraction buffer, and collected by centrifugation at 3000*g*. Subsequently, the immunoprecipitants were subjected to western blot.

### Celigo cell counting assay

AGS and MGC-803 cells were transfected with lentivirus and cultured in 96-well plates at a density of 2000 cells per well. The cells were counted at 24, 48, 72 and 96 h by Celigo (Nexcelom) and the cell growth curve was plotted for the 5 days.

### Colony formation assay

MGC-803 cells were transfected with lentivirus and cultured in six-well plates at a density of 1000 cells per well. Cell colonies were washed twice by using cold phosphate buffered saline (PBS), fixed with 75% ethanol and stained with 0.1% crystalline purple. They were cultured until the colonies were visible, counted and photographed.

### Cell apoptosis detection

AGS and MGC-803 cells were transfected with lentivirus and inoculated into six-well plates (2 mL/well) for 5 days. The cells were centrifuged for 5 min, the cell precipitates were successively eluted with precooled D-hanks (pH = 7.2–7.4) and 1×binding buffer (eBioscience), resuspended by 200 µL 1× binding buffer, stained with 10 µL Annexin V-APC (eBioscience) at room temperature in the dark for 15 min and detected by flow cytometry (Guava easyCyte HT, Millipore).

### Transwell assay

Transwell chambers (24-well, 8-mm pore, MA, USA) were used to measure the migration ability of the cells in 24-well plates. AGS and MGC-803 cells were transferred into the upper chamber with 200 µL serum-free medium and medium with 30% FBS was added to the lower chamber. The cells on the upper chamber were removed, while the cells adhering to the Polycarbonate membrane were fixed with 4% precooled paraformaldehyde for 30 min and stained with 0.1% crystal violet for 20 min at room temperature. Finally, the migrated cells were photographed from five randomly selected fields under a 200 × microscope.

### Wound-healing assay

AGS and MGC-803 cells were cultured into 6-well plates (100 µL/well) at a density of 4000 cells per well. The cells were eluted with PBS, fixed with 3.7% paraformaldehyde (Corning) for 15 min, stained with 1% crystal violet for 10 min. Wound healing was observed at 0 h, 8 and 72 h under a microscope for image acquisition and Image J software (National Institutes of Health) was used to quantify the distance (µm) between the scratches.

### Sphere-forming assay

Sphere-forming ability is an important method for the identification of tumor stem cells in vitro, and is indicated by the Sphere Formation Efficiency (SFE). AGS and MGC-803 cells balls were collected and cultured by 70 μm cell sieve for 10–14 days and digested into single cells using trypsin. An appropriate number of cells were spread into 96-well plates for further culture for 10–14 days, the number of cell balls was counted, and SFE was calculated by the formula: SFE = Number of cell balls with diameter greater than 75 μm per well/total number of original inoculated cells per well.

### Xenograft tumor assay

BALB/c nude mice (age, 4–6 weeks; weight, 16–20 g) were ordered from the animal laboratory center (Vitong Lihua, Beijing). The experimental procedures performed on mice were approved by the Ethics Committee of Henan Tumor Hospital and the Animal Protection Association. MGC-803 cells transfected with lentivirus Control or PHF5A were subcutaneously injected into the right armpit of mice, respectively (4 × 10^6^ cells/mouse). Subsequently, mice in PHF5A group were given intravenous Pladienolide B at a daily dose of 5 mg/kg and kept fed normally. A week after injection, tumor volume was monitored 1 to 2 times a week and calculated using the following formula: Volume (mm^3^) =π/6 × L × W × W (L: longest dimension, W: dimension perpendicular to length). In addition, the mice were anesthetized by intraperitoneal injection of 0.7% pentobarbital sodium at a dose of 10 µL/g, and tumor burden was observed (emission wavelength, 510 nm) under in vivo imager system (Perkin Elmer, IVIS Spectrum). After 24 days, mice were sacrificed by cervical dislocation and the tumors were subjected to detection of PHF5A, Ki67 expression using IHC staining.

### Microarray analysis

RNA was purified from MGC-803 cells transfected with lentivirus shCtrl or shPHF5A, and analyzed by A Affymetrix Human Gene Microarray Prime View (Affymetrix Scanner 3000 scan) to identify differentially expressed genes (DEGs). The screening criteria of DEGs was |Fold Change| ≥ 1.3 and false discovery rate (FDR) ≤ 0.05. Significant enrichment of DEGs in classical pathways, disease and function, and interaction networks was explored based on Ingenuity Pathway Analysis (IPA).

### Statistical analysis

Data were obtained from three independent experiments which are presented as the means ± standard deviation (SD) and a P-value < 0.05 was considered significant. Comparisons between different groups were analyzed with the *t* test. The significance of differences between groups was assessed by GraphPad Prism V8.0 (GraphPad, CA, USA) and SPSS 20.0 (IBM, SPSS, IL, USA).

## Results

### PHF5A is abundantly expressed in GC

**Fig. 1:**
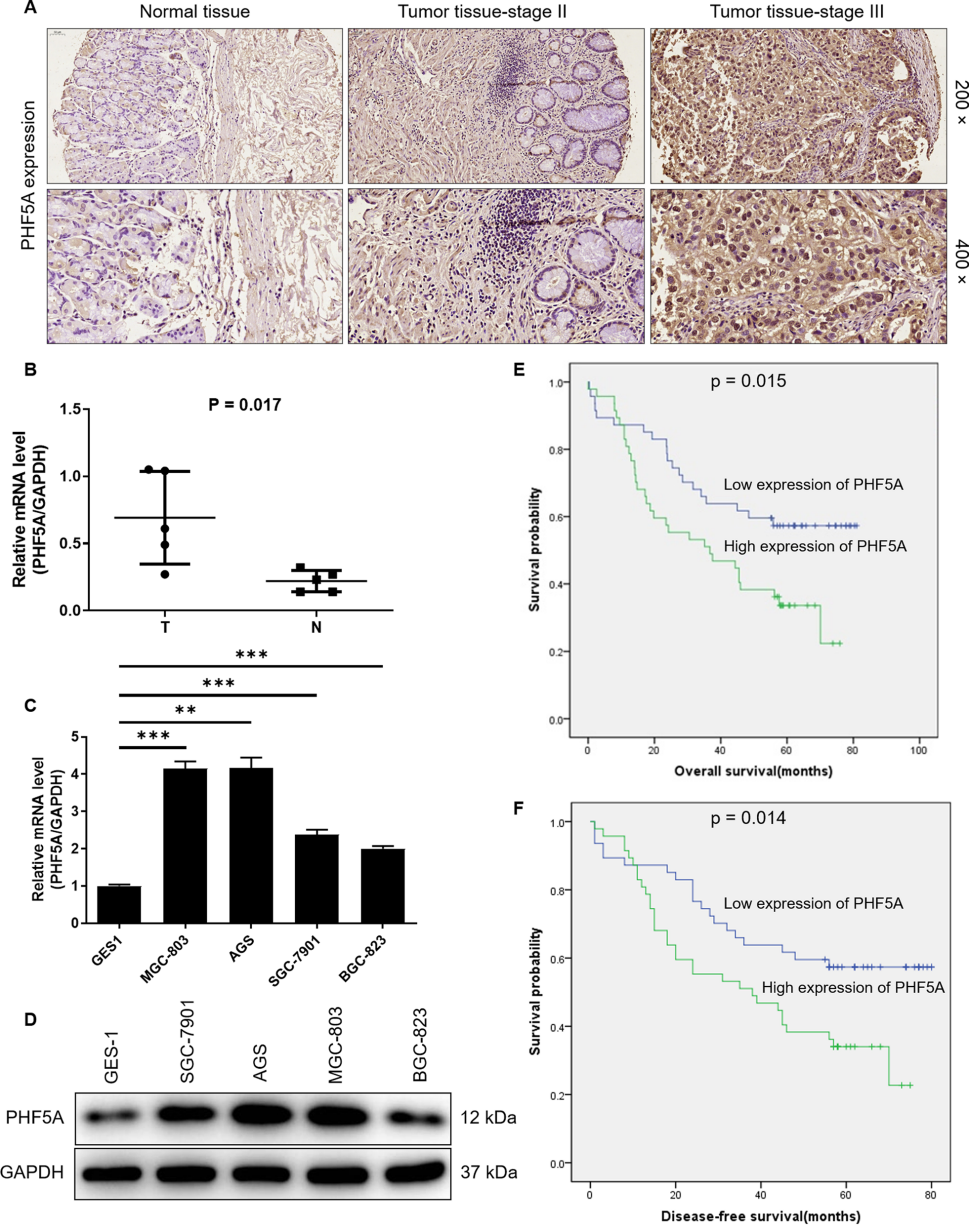
The high expression of PHF5A in GC exerts clinical value for poor prognosis.** A** PHF5A was highly expressed in tumor tissues compared with adjacent normal tissues in GC patients. (The magnification is 200× and 400×). **B** The mRNA level of PHF5A in GC tumor tissues and normal tissues. **C**, **D** The mRNA and protein levels of PHF5A in human GC cell lines MGC-803, AGS, SGC-7901, BGC-823 and normal gastric mucosa cells GES1cells. The presented results were representative of experiments repeated at least three times. Data was represented as mean ± SD. ***P < 0.001. **E**, **F** Correlation between PHF5A expression level and overall survival/disease-free survival (months) in patients with GC

Firstly, PHF5A expression profile in GC patients were conducted based on TCGA database, indicating that the expression of PHF5A in GC was quite abundant, but not significant (P = 0.432) (Additional file 1: Fig. S1A). Furthermore, the expression of PHF5A was assessed by immunohistochemical staining in GC patients of tumor tissues and adjacent normal tissues, and our results showed that the expression of PHF5A was significantly elevated in GC tissues (59/114, 51.2%) compared with adjacent normal tissues (P < 0.001; Table 1). The representative images of the immunohistochemical staining indicated that PHF5A signal intensity in GC tissues was indeed stronger than that in adjacent normal tissues (Fig. 1A). Consistently, the data of qRT-PCR showed that the mRNA level of PHF5A in GC tumor tissue was significantly higher than that in normal tissue (P = 0.017; Fig. 1B). Moreover, the mRNA and protein levels of PHF5A in human GC cell lines MGC-803, AGS, SGC-7901, BGC-823 were significantly higher than those in normal gastric mucosa cells GES1cells (P < 0.001; Fig. 1C, D). Collectively, PHF5A was abundantly expressed in GC tissues and cells.

**Table 1:** Expression patterns in gastric cancer tissues and para-carcinoma tissues revealed in immunohistochemistry analysis

PHF5A expression	Tumor tissue		Normal tissue		P value
	Cases	Percentage	Cases	Percentage	
Low	55	48.8%	103	92.0%	< 0.0001
High	59	51.2%	9	8.0%	

### Correlation between the expression level of PHF5A and the pathological characteristics of GC

The relationships between PHF5A expression and clinic characteristics of GC were analyzed according to Mann-Whitney U, the expression of PHF5A was significantly positively correlated with the pathological data such as gender (P = 0.048), T Infiltrate (P = 0.047) and pathological stage (P = 0.042) (Table 2). Furthermore, multivariate statistical analysis was performed based on the TCGA database to reveal the correlation between PHF5A expression and available features in clinicopathologic GC patients. The results of univariable and multivariate analyses indicated that patient age at diagnosis (P = 0.036), TNM stage (P = 0.001), Pathologic_T (P = 0.017), Pathologic_N (P = 0.006) and Pathologic_M (P = 0.006) were significantly associated with prognosis of GC patients by univariate analysis. Furthermore, the multivariate analysis showed that patient age at diagnosis (P = 0.006), Pathologic_N (P = 0.077) and Pathologic_M (P = 0.007) served as significantly and independently prognostic factors for overall survival rate of GC patients by the Cox proportional hazards regression model, whereas the PHF5A expression (P > 0.05 for each) failed to provide independently prognostic value (Table 3). By Kaplan-Meier survival analysis based on the TCGA database, founding that the expression level of PHF5A had no significant effect on the survival time of GC patients (P = 0.37; Additional file 1: Fig. S1B). Moreover, the survival correlation analysis was performed again based on the clinical follow-up information we collected. As showed in Fig. 1E, F, the expression level of PHF5A was significantly related to overall survival (P = 0.015) and disease-free survival (P = 0.014) of GC. As the expression of PHF5A in patients increased, the survival period was significantly shortened.

**Table 2:** Relationship between PHF5A expression and tumor characteristics in patients with gastric cancer

Features	No. of patients	PHF5A expression		P value
		Low	High	
All patients	114	55	59	
Age (years)				0.260
≤ 64	58	31	27	
> 64	56	24	32	
Gender				0.048
Male	79	43	36	
Female	35	12	23	
Tumor size				0.889
< 5 cm	48	23	25	
≥ 5 cm	58	27	31	
Differentiation				0.814
Low	41	19	22	
Mediate	57	29	28	
High	3	1	2	
T Infiltrate				0.047
T1	6	4	2	
T2	12	8	4	
T3	73	35	38	
T4	23	8	15	
lymphatic metastasis (N)				0.182
N0	18	10	8	
N1	16	10	6	
N2	26	12	14	
N3	54	23	31	
Stage				0.042
I	8	5	3	
II	30	18	12	
III	74	32	42	
IIII	2	0	2	
Vessel carcinoma embolus				0.603
0	25	13	12	
1	59	27	32	
Nerve tumor infiltrates				0.819
0	30	14	16	
1	20	10	10	
Expression of Ki67				0.124
< 60%	44	25	19	
≥ 60%	58	24	34	
Expression of CD34				0.744
No	13	6	7	
Yes	31	16	15	
Expression of EGFR				0.650
No	87	43	44	
Yes	14	6	8	
Expression of VEGF				0.779
No	41	19	22	
Yes	61	30	31	
Expression of CDX2				0.281
No	18	11	7	
Yes	85	40	45	
Expression of Her2				0.954
No	75	37	38	
Yes	26	13	13	
Number of lymphatic metastases				0.134
≤ 6	58	32	26	
> 6	56	23	33	

**Table 3:** Univariate and multivariate analyses of various potential prognostic factors in GC patients by the cox proportional hazards regression model

Variables	Group	Univariate analysis		Multivariate analysis		
		HR		P val.	HR	P val.
CENPO Expression	High (> 1355)	1			1	
	Low ( < = 1355)	1.156(0.836–1.598)		0.38	1.4(0.981–1.998)	0.064
Age_at_diagnosis	>=67	1			1	
	< 67	0.705(0.509–0.978)		0.036	0.595(0.412–0.861)	0.006
Gender	Female	1			1	
	Male	1.311(0.923–1.862)		0.13	1.452(0.997–2.115)	0.052
Stage	T1/2	1			1	
	T3/4	1.83(1.287–2.603)		0.001	1.096(0.62–1.935)	0.753
Pathologic_T	T1/2	1			1	
	T3/4	1.653(1.095–2.495)		0.017	1.496(0.894–2.503)	0.125
Pathologic_N	N0/1	1			1	
	N2/3	1.587(1.142–2.204)		0.006	1.538(0.955–2.478)	0.077
Pathologic_M	M0	1			1	
	M1	2.186(1.257–3.803)		0.006	2.388(1.265–4.511)	0.007

h: Hazard ratio

### Downregulation of PHF5A inhibits the malignant phenotypes of GC

**Fig. 2:**
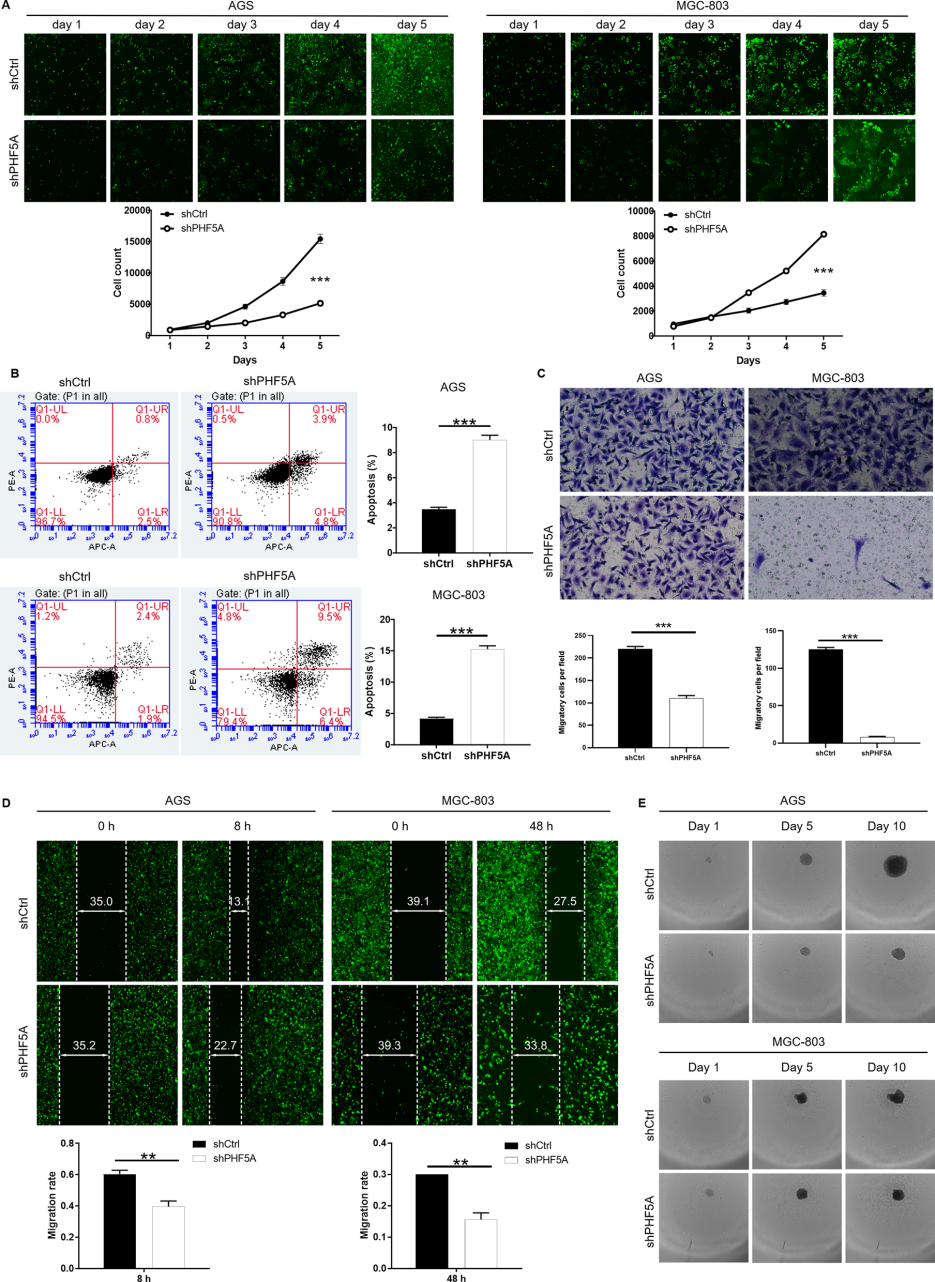
Knockdown down of PHF5A acts as a tumor suppressor in GC cells.** A** The effect of reduced PHF5A expression on the proliferation of AGS and MGC-803 cells was estimated by Celigo cells counting assay. **B** Flow cytometry was used to estimate the effect of reduced PHF5A expression on the apoptosis ability of AGS and MGC-803 cells. **C**, **D** Transwell and wound-healing experiments were performed to evaluate the effect of reduced PHF5A expression on AGS and MGC-803 cell migration. **E** Effect of PHF5A knockdown on GC stem cells was evaluated by Sphere-forming assay. The presented results were representative of experiments repeated at least three times. Data was represented as mean ± SD. **P < 0.01, ***P < 0.001

To investigate the function of PHF5A in GC, the expression of PHF5A in the AGS, MGC-803 cell lines was knocked down using a specific siRNA (shPHF5A) and shCtrl as a control. The interfering, specifically targeting human PHF5A (shPHF5A-1/2/3) were synthesized, and shPHF5A-3 group had the highest knockdown efficiency (71.4%) compared with other groups (P < 0.01; Additional file 1: Fig. S1C). Subsequently, qRT-PCR results showed that knockdown efficiency of PHF5A was 50.20% and 50.04% in AGS (P < 0.01) and MGC-803 (P < 0.001) compared with shCtrl group. In addition, results from western blot analysis verified that the protein level of PHF5A in shPHF5A group was downregulated compared to shCtrl group (Additional file 1: Fig. S1D). Five days of culture and counting of AGS and MGC-803 showed that the cells number in the shPHF5A group was significantly lower than that in the shCtrl group (P < 0.001; Fig. 2A). Results from flow cytometry analysis demonstrated that the apoptosis rate of AGS and MGC-803 cells in the shPHF5A group was 2.6 times higher than that in the shCtrl group (P < 0.001; Fig. 2B). Moreover, Transwell assays showed that cell mobility in AGS and MGC-803 cells was reduced by 50% and 94% in the shPHF5A group compared with the shCtrl group, respectively (P < 0.001; Fig. 2 C). Consistently, limited migration of AGS and MGC-803 cells was observed in the PHF5A low expression group compared to the controls in wound healing during 8–48 h (P < 0.01; Fig. 2D). In addition, the sphere-forming ability of AGS and MGC-803 cells in shPHF5A was weaker than that in the control group, suggesting that PHF5A knockdown inhibited the formation of GC stem cells (Fig. 2E). The AKT/mammalian target of rapamycin (mTOR) signaling pathway is essential for cell growth and survival in cancer[23, 24]. GC cells after PHF5A knockdown showed that the phosphorylation level of AKT and mTOR decreased, and the addition of AKT activator could partially reverse this effect (Additional file 1: Fig. S1E). In addition, the treatment of AKT activator weakened the promoting effect of PHF5A knockdown on GC cell apoptosis (Additional file 1: Fig. S1F). Collectively, these results suggested that knockdown of PHF5A could inhibit progression of GC through AKT/mTOR signaling pathway.

### Knockdown of PHF5A promotes ubiquitination of FOS through the E3 ubiquitin ligase SKP2

**Fig. 3:**
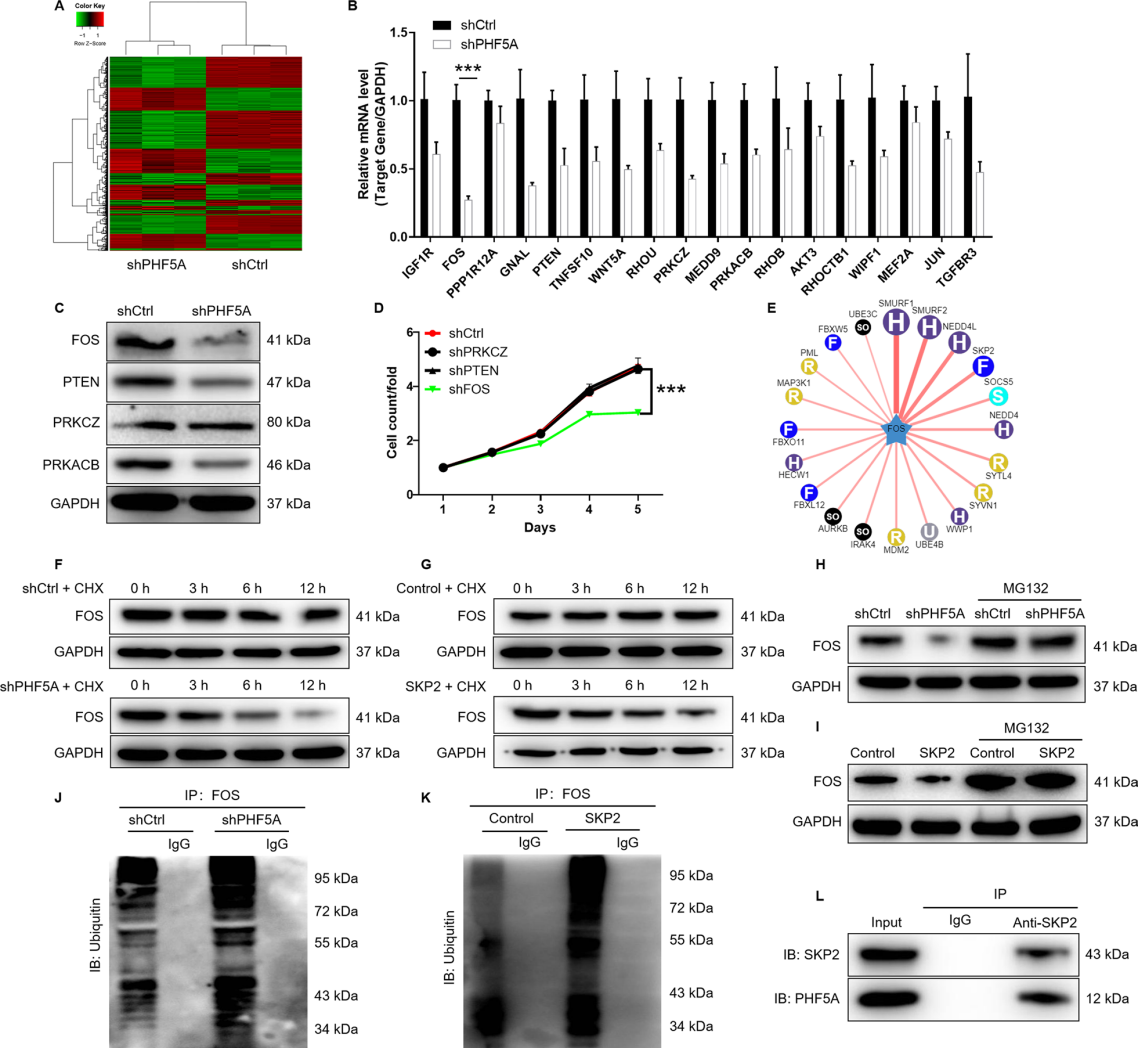
PHF5A regulates the protein stability of FOS through SKP2-mediated ubiquitination.** A** Prime View Human Gene Expression Array was performed to identify DEGs between shCtrl and shPHF5A groups of MGC-803 cells. In the heat map of cluster analysis, each column represents a sample and each row represents a differential gene. The red indicates that the gene expression is relatively up-regulated, the green indicates that the gene expression is relatively down-regulated, the black indicates that the gene expression is not significantly changed, and the gray indicates that the signal strength of the gene is not detected. **B**, **C** The most significant DEGs in MGC-803 cells with PHF5A knockdown were screened by qRT-PCR and western blot analysis. **D** The effect of FOS, PTEN, PRKCZ knockdown on proliferation was detected using Celigo cell counting assay. **E** The E3 ubiquitin ligase affecting FOS protein stability was predicted by bioinformatics analysis. **F**, **G** The protein stability of FOS in MGC-803 cells after PHF5A knockdown and SKP2 overexpression was examined. **H**, **I** After treatment with MG-132, the protein stability of FOS in MGC-803 cells after PHF5A knockdown and SKP2 overexpression was examined. **J**, **K** The lysates of MGC-803 cells after PHF5A knockdown and SKP2 overexpression were immunoprecipitated and WB was performed to examine the ubiquitination of FOS. **L** Co-IP analysis of interaction of PHF5A and SKP2 in MGC-803 cells. The presented results were representative of experiments repeated at least three times. Data was represented as mean ± SD. ***P < 0.001

MGC-803 cells transfected with lentivirus shCtrl or shPHF5A were subjected to microarray analysis to identify DEGs. The results indicated that knockdown of PHF5A resulted in upregulation of 1392 genes and downregulation of 1851 genes in MGC-803 cells, which were presented in the Fig. 3A. According to IPA, we found that these DEGs were enriched in classic signaling pathways, disease and function, such as nuclear factor kappa B (NF-κB) signaling pathway, activation of sumoylation pathway, cancer, cell death and survival (Additional file 1: Fig. S2A, B). Subsequently, the most significant DEGs were further screened by qRT-PCR (Fig. 3B) and western blot analysis (Fig. 3C), indicating that FOS was the most significantly downregulated gene. Moreover, lentivirus was used to deliver shRNAs into MGC-803 cells to knockdown FOS, PTEN, PRKCZ, respectively. Compared with other groups, MGC-803 with FOS knockdown showed the strongest inhibitory effect on proliferation (P < 0.001; Fig. 3D), suggesting that FOS was relevant to the proliferative ability of GC cells. In addition, the results of IPA showed the interaction network between PHF5A and FOS (Additional file 1: Fig. S2C). Thus, the mechanism of PHF5A regulating GC and the relationship between PHF5A and FOS had attracted our attention.

Previous study reported that the protein stability of FOS was regulated by E3 ligase UBR1-mediated ubiquitination[25]. Through bioinformatics analysis (http://ubibrowser.ncpsb.org.cn/ubibrowser/strict/networkview/networkview/name/P01100/jobId/ubibrowse-I2021-09-10-57171-1631252382), we predicted that the protein stability of FOS was indeed affected by multiple E3 ubiquitin ligases (Fig. 3E). Moreover, microarray results showed that S-phase kinase-associated protein 2 (SKP2) was significantly differentially expressed gene among the DEGs after the knockdown of PHF5A. As a consequence, we hypothesized that PHF5A regulated the protein stability of FOS through SKP2 mediation. In order to verify our hypothesis, we carried out the following experiments. The MGC-803 cells with PHF5A knockdown or SKP2 overexpression were treated with protein synthesis inhibitor CHX (0.2 mg/mL) to test the stability of FOS protein. The downregulation of PHF5A accelerated the protein degradation of FOS (Fig. 3F). Meanwhile, SKP2 overexpression also reduced the protein stability of FOS (Fig. 3G). The results indicated that the protein stability of FOS was affected by HF5A and SKP2. After treatment with proteasome inhibitor MG-132 (20 µM), the effect of PHF5A knockdown and SKP2 overexpression on the stability of FOS protein could be partially eliminated (Fig. 3 H, I). We inferred that PHF5A and SKP2 regulated FOS through ubiquitin-proteasome system (UPS). Subsequently, we evaluated the regulation of PHF5A and SKP2 on ubiquitination of FOS. The results suggested that PHF5A downregulation and SKP2 overexpression distinctly promoted FOS ubiquitination (Fig. 3J, K). Furthermore, the results of Co-IP assay revealed that there is an interaction between PHF5A and E3 ligase SKP2 (Fig. 3L). Taken together, PHF5A regulated the protein stability of FOS through SKP2-mediated ubiquitination.

### Downregulation of FOS attenuates the promotion of PHF5A overexpression on GC cells

**Fig. 4:**
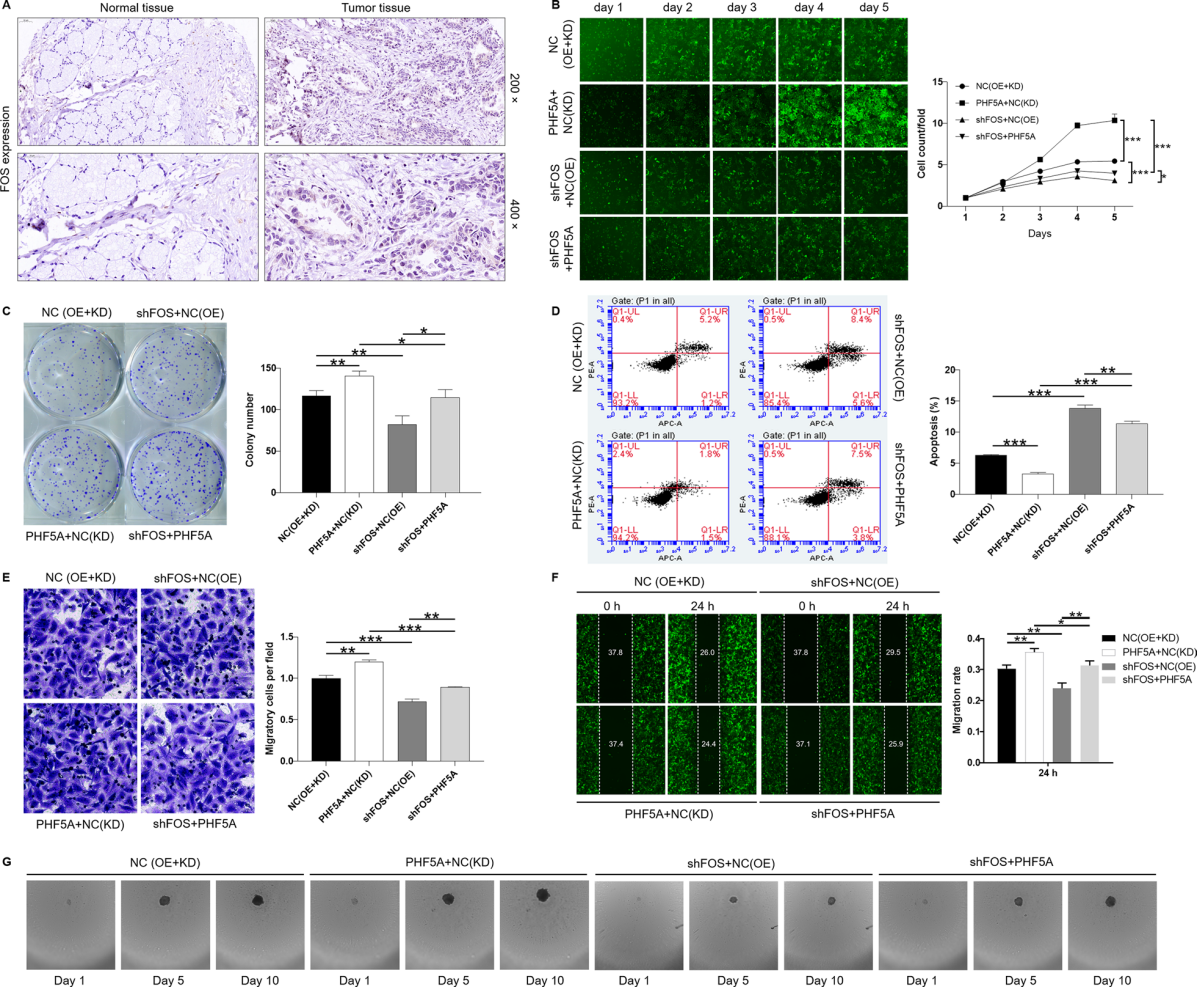
Downregulation of FOS attenuates the promotion of PHF5A overexpression on GC cells. **A** FOS was highly expressed in tumor tissues compared with adjacent normal tissues in GC patients. (The magnification is 200 × and 400 ×). **B** Cell proliferation in MGC-803 was measured using Celigo cell counting assay. Results were normalized to viability on day 1 and represented as fold change. **C** The clone number of MGC-803 cells was estimated by colony formation assay. **D** Apoptosis ability of MGC-803 cells was analyzed using Annexin V-APC staining, followed by flow cytometry. **E**, **F** Migration of MGC-803 cells was determined by Wound healing assay and Transwell assay. **G** Effect of PHF5A knockdown on MGC-803 cells stem was evaluated by Sphere-forming assay. The presented results were representative of experiments repeated at least three times. Data was represented as mean ± SD. *P < 0.05, **P < 0.01, ***P < 0.001

The expression level of FOS in GC was confirmed by immunohistochemical staining, and the results showed that FOS expression in tumor tissue was significantly higher than that in normal tissues (Fig. 4A). In addition, FOS was highly expressed in GC cell lines AGS, BGC-823, MGC-803, SGC-7901 (Additional file 1: Fig. S2D). Subsequently, FOS knockdown MGC-803 cells were established to explore the effect of FOS on the phenotype of GC (P < 0.01) (Additional file 1: Fig. S2E). As expected, FOS-knocked-down [shFOS + NC(OE)] MGC-803 cells showed a significant inhibition of proliferation (P < 0.001), colony formation (p < 0.01), migration (P < 0.01), sphere-forming, and a significant promotion of apoptosis (P < 0.001; Fig. 4B–F). Not surprisingly, PHF5A overexpression [PHF5A + NC(KD)] could promote the malignant progression of GC cells (P < 0.01; Fig. 4B–F). Furthermore, we transfected lentiviruses with a combination of FOS low expression and PHF5A overexpression (shFOS + PHF5A) to further detect the alteration of GC cells. The loss/gain-of-assays indicated that downregulation of FOS attenuated the promotion of PHF5A overexpression on GC cells (Fig. 4B–F). Taken together, PHF5A promoted GC progression, which could be partially reversed by knockdown of FOS.

### Pladienolide B treatment reverses the induction of PHF5A overexpression on the progression of GC cells

**Fig. 5:**
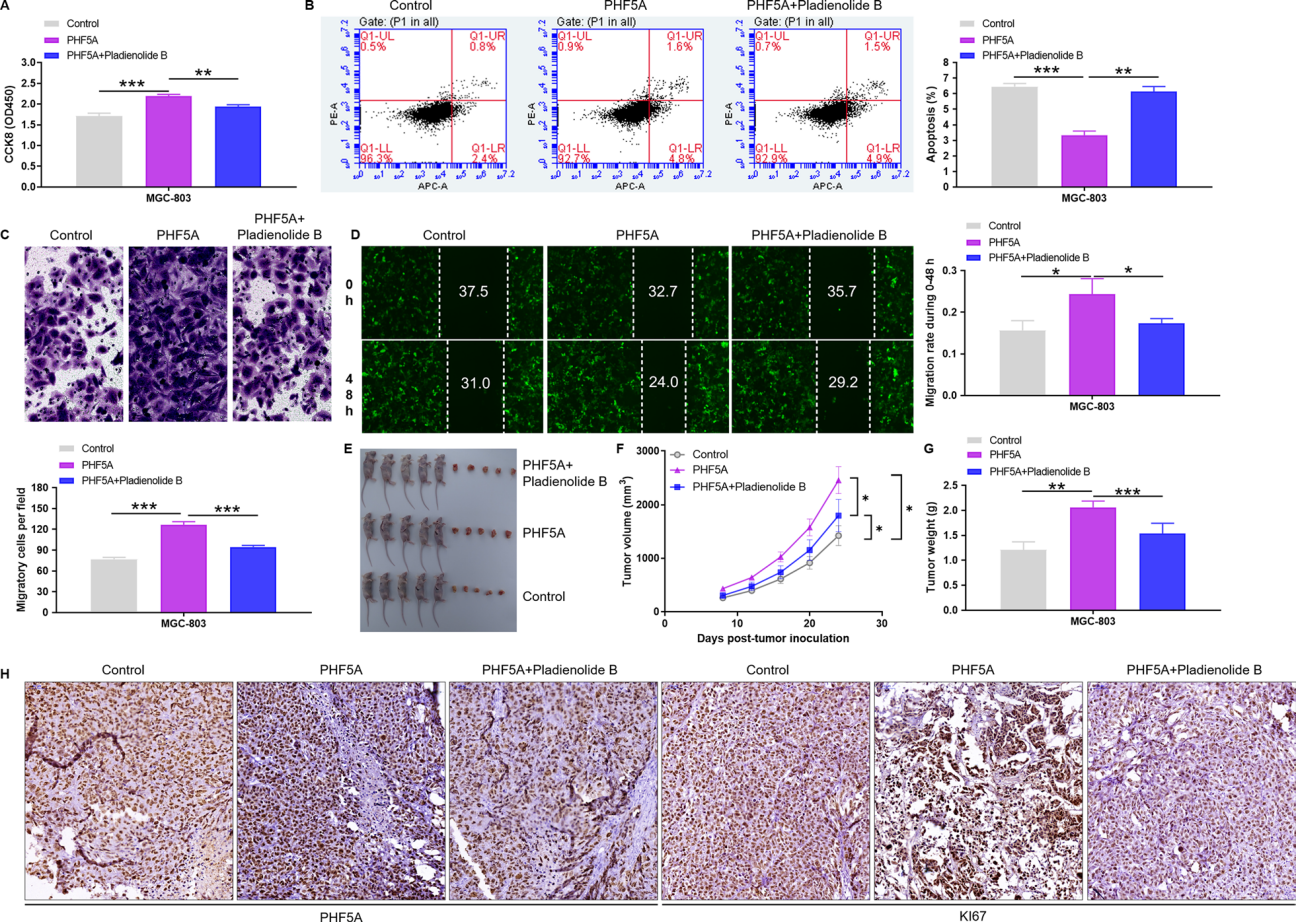
Pladienolide B treatment reverses the induction of PHF5A overexpression on the progression of GC cells.** A–D** PHF5A overexpressed cells were treated with Pladienolide B to detect changes in cell viability, apoptosis, and migration. Notably, for Figure **D**, **H** is the migration time of the cell. The presented results were representative of experiments repeated at least three times. Data was represented as mean ± SD. *P < 0.05, **P < 0.01, ***P < 0.001. **E–G** PHF5A overexpression and control MGC-803 cells were subcutaneous injected into nude mice followed by intravenous Pladienolide B treatment to observed tumor volume, tumor size and immunohistochemical staining. Data was represented as mean ± SD (n = 5). *P < 0.05, **P < 0.01, ***P < 0.001

Pladienolide B is a small molecule reported to be a PHF5A inhibitor [26]. GC cells were treated with the Pladienolide B and tested in vitro functions (proliferation, apoptosis, migration, wound healing) and in vivo tumorigenesis. In vitro functional verification, PHF5A overexpression significantly promoted the malignant phenotype of GC cells, such as the enhancement of cell viability (P < 0.001; Fig. 5A) and migration (P < 0.05; Fig. 5B, C), and the decrease of apoptosis rate (P < 0.001; Fig. 5D). Not surprisingly, the addition of Pladienolide B attenuated this effect (Fig. 5A–D). In vivo heterograft tumor monitoring for 24 days, it was found that overexpression of PHF5A significantly promoted the tumor formation of GC cells, which was manifested by increased tumor size (P < 0.05; Fig. 5E, F), weight (P < 0.01; Fig. 5G) and KI67 expression (Fig. 5H). Moreover, consistent with in vitro results, Pladienolide B attenuated the tumor formation effect caused by PHF5A overexpression (Fig. 5E–H).

## Discussion

In this study, the role of PHF5A in promoting the malignant progression of GC was clarified. We identified that PHF5A expression was remarkably upregulated in GC. In addition, the relationship between the expression of PHF5A and clinicopathological features in GC was analyzed. Previous study reported that tumor cells infiltrating T lymphocytes indicated the malignant progression of cancer [27]. Consistently, the present study clarified that high expression of PHF5A was positively associated with T Infiltrate and pathological stage in GC patients. Moreover, high expression of PHF5A usually predicted the shortening of the survival time of patients with GC. Therefore, PHF5A expression level was related to the malignant progression and prognosis of GC, which exerted important clinical value.

Furthermore, in vitro loss-of-function assays demonstrated that knockdown of PHF5A inhibited the biological phenotypes of GC cells, which showed weakened proliferation, enhanced apoptosis and suppression of migration. Previous studies have shown that PI3K/AKT/mTOR signaling pathway plays a key role in the signal transduction, apoptosis, proliferation, cell cycle and growth of tumor cells [28–30]. In this study, we examined the effect of PHF5A knockdown on the expression of AKT/mTOR pathway proteins. GC cells after PHF5A knockdown showed that the phosphorylation level of AKT and mTOR decreased, and the addition of AKT activator could partially reverse this effect. In addition, the treatment of AKT activator weakened the promoting effect of PHF5A knockdown on GC cell apoptosis. Collectively, these results suggested that knockdown of PHF5A could inhibit progression of GC through AKT/mTOR signaling pathway.

Furthermore, the results of microarray analysis preliminarily showed that FOS is the downstream target of PHF5A in regulating GC. The FOS proteins have been implicated as regulators of cell proliferation, differentiation, and transformation [31]. FOS is ubiquitous in a variety of cancers and is a powerful driving force for tumor development [32, 33]. Moreover, FOS involvement in the development of multidrug resistance in some tumors, such as breast and ovarian cancer [34, 35]. However, the mechanism of FOS regulation of cancer has not been fully explained.

Previous study reported that the protein stability of FOS was regulated by E3 ligase UBR1-mediated ubiquitination [25]. Ubiquitination is a highly regulated and reversible post-translational protein modification that degrades or regulates protein function via the ubiquitin-proteasome system (UPS) [36]. In the UPS, the E3 ligase SKP2 could interact with c-Myc and participate in its ubiquitination and proteasome degradation [37, 38]. A large number of studies have shown that ubiquitination plays an important role in the progression of cancers and have revealed the great therapeutic potential of targeted ubiquitination in a variety of cancers [39–42]. Here, we uncovered a new regulatory mechanism of PHF5A in GC and found that PHF5A regulated the protein stability of FOS through SKP2-mediated ubiquitination. Moreover, the present study clarified that downregulation of FOS attenuated the promotion of PHF5A overexpression on GC cells. Taken together, knockdown of PHF5A inhibited the progression of GC through SKP2-mediated ubiquitination of FOS.

## Supplementary Information

**Additional file 1: Fig. S1. **A. PHF5A expression profile in GC patients were conducted based on TCGAdatabase. B. A correlation analysis of Kaplan-Meier survival and PHF5Aexpression in GC patients was performed based on the TCGA database. C. The expression level of PHF5A was detected in 3different shRNAs against PHF5A sequences. D. Thespecificity and validity of the lentivirus-mediated shRNA knockdown of PHF5Aexpression was verified by qPCR and WB. E. Detection of AKT and mTOR phosphorylation levels oflentivirus shPHF5A transfected MGC-803 cells after treatment with AKTactivator. F. Detection of apoptosis ability of MGC-803 cells after treatment withAKT activator. The representative images were selected from atleast 3 independent experiments. Data was represented as mean ± SD. *P < 0.05, **P < 0.01, ***P< 0.001.  **Fig. S2. **A. The enrichment of the DEGsin IPA disease and function was analyzed by IPA. B. Theenrichment of the DEGs in canonical signaling pathways was analyzed by IPA. C. Interactionnetwork diagram among DEGs was analyzed by IPA. D. The mRNA expression level of FOS in human GCcell lines AGS, BGC-823, MGC-803, SGC-7901. E. The expression level of FOS was detected in 3different shRNAs against FOS sequences. The representative images were selected from atleast 3 independent experiments. Data was represented as mean ± SD. *P <0.05, **P < 0.01, ***P < 0.001. **Table S1.** The targetsequences and shRNA sequences.

**Additional file 2: Table S2.** Primers used in qPCR.

**Additional file 3: Table S3.** Antibodies used in western blotting and Co-IP.

## Data Availability

All data analysis is showed in this article.
