# Supplementary material for: PHF5A facilitates the development and progression of gastric cancer through SKP2-mediated stabilization of FOS
Source: J Transl Med. 2023 Jan 6;21:5. doi: 10.1186/s12967-022-03821-w (PMC9817416; doi:10.1186/s12967-022-03821-w)
Supplement: Supplementary file 1 — Additional file 1: Fig. S1. A. PHF5A expression profile in GC patients were conducted based on TCGAdatabase. B. A correlation analysis of Kaplan-Meier survival and PHF5Aexpression in GC patients was performed based on the TCGA database. C. The expression level of PHF5A was detected in 3different shRNAs against PHF5A sequences. D. Thespecificity and validity of the lentivirus-mediated shRNA knockdown of PHF5Aexpression was verified by qPCR and WB. E. Detection of AKT and mTOR phosphorylation levels oflentivirus shPHF5A transfected MGC-803 cells after treatment with AKTactivator. F. Detection of apoptosis ability of MGC-803 cells after treatment withAKT activator. The representative images were selected from atleast 3 independent experiments. Data was represented as mean ± SD. *P < 0.05, **P < 0.01, ***P< 0.001. Fig. S2. A. The enrichment of the DEGsin IPA disease and function was analyzed by IPA. B. Theenrichment of the DEGs in canonical signaling pathways was analyzed by IPA. C. Interactionnetwork diagram among DEGs was analyzed by IPA. D. The mRNA expression level of FOS in human GCcell lines AGS, BGC-823, MGC-803, SGC-7901. E. The expression level of FOS was detected in 3different shRNAs against FOS sequences. The representative images were selected from atleast 3 independent experiments. Data was represented as mean ± SD. *P <0.05, **P < 0.01, ***P < 0.001. Table S1. The targetsequences and shRNA sequences. [file 12967_2022_3821_MOESM1_ESM.docx]

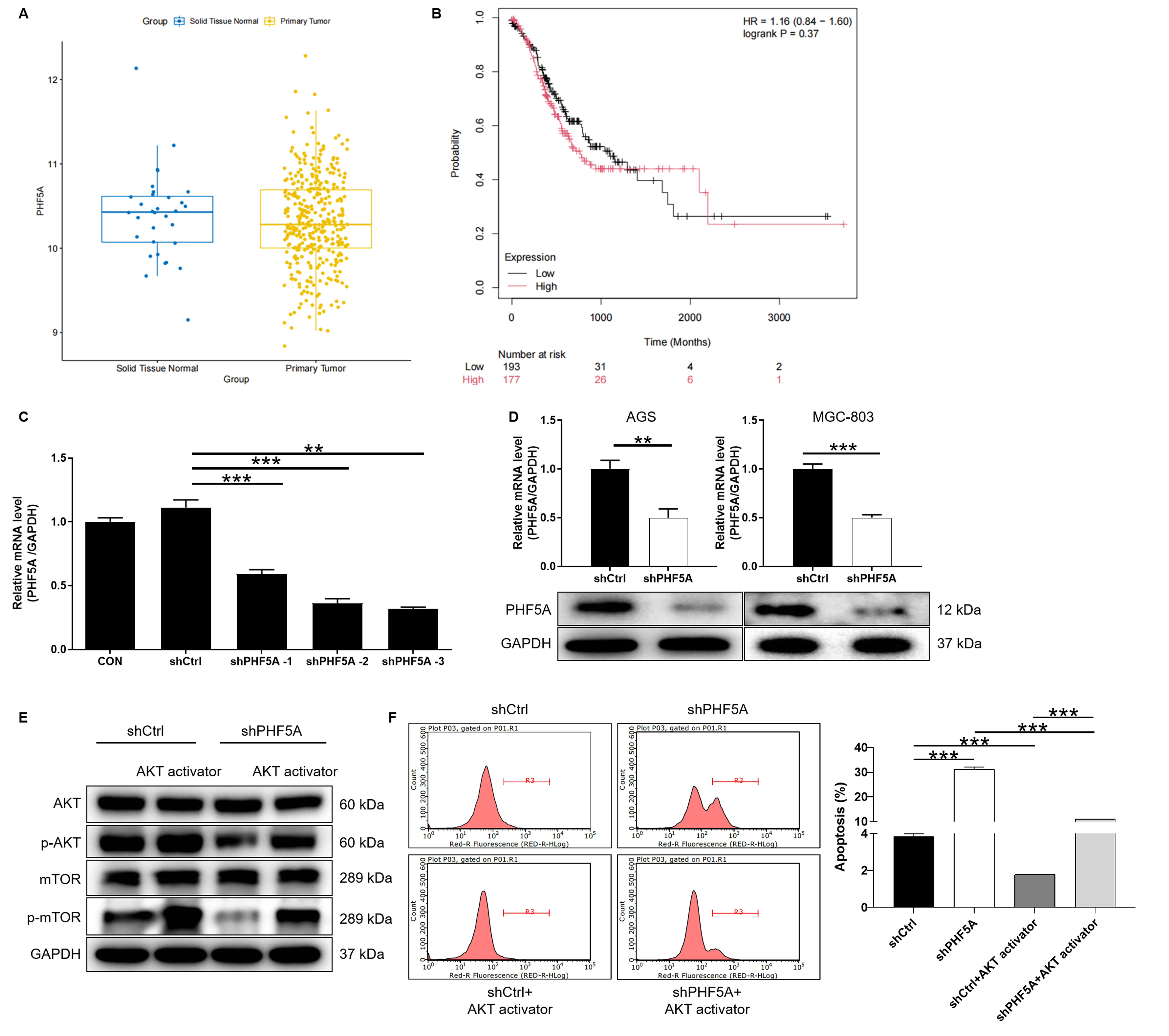


**Fig. S1. A.** PHF5A expression profile in GC patients were conducted based on TCGA database. **B.** A correlation analysis of Kaplan-Meier survival and PHF5A expression in GC patients was performed based on the TCGA database. **C.** The expression level of PHF5A was detected in 3 different shRNAs against PHF5A sequences. **D.** The specificity and validity of the lentivirus-mediated shRNA knockdown of PHF5A expression was verified by qPCR and WB. **E.** Detection of AKT and mTOR phosphorylation levels of lentivirus shPHF5A transfected MGC-803 cells after treatment with AKT activator. **F.** Detection of apoptosis ability of MGC-803 cells after treatment with AKT activator. The representative images were selected from at least 3 independent experiments. Data was represented as mean ± SD. *P < 0.05, **P < 0.01, ***P < 0.001.


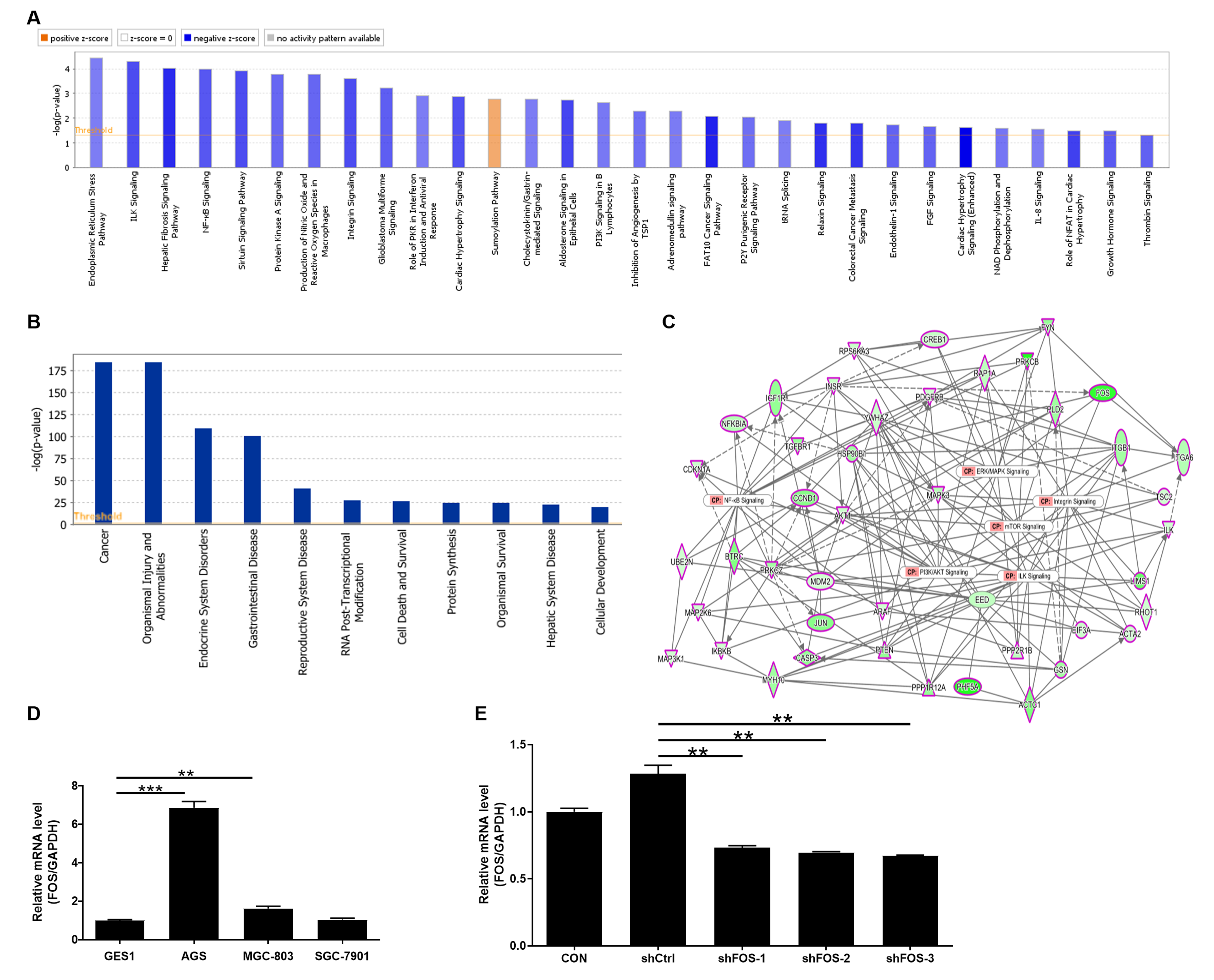


**Fig. S2 A.** The enrichment of the DEGs in IPA disease and function was analyzed by IPA. **B.** The enrichment of the DEGs in canonical signaling pathways was analyzed by IPA. **C.** Interaction network diagram among DEGs was analyzed by IPA. **D.** The mRNA expression level of FOS in human GC cell lines AGS, BGC-823, MGC-803, SGC-7901. **E.** The expression level of FOS was detected in 3 different shRNAs against FOS sequences. The representative images were selected from at least 3 independent experiments. Data was represented as mean ± SD. *P < 0.05, **P < 0.01, ***P < 0.001.

Table S1. The target sequences and shRNA sequences.

| Gene | shRNA sequences (5'-3') |
| --- | --- |
| Scramble sequence (shCtrl) | TTCTCCGAACGTGTCACGT |
| shPHF5A-1 | GCGCATATGTGATGAGTGTAA |
| shPHF5A-2 | TAAGACAGACCTCTTCTATGA |
| shPHF5A-3 | AATGTGATGGCAAGTGTGTGA |
| shFOS-1 | GTGGAACAGTTATCTCCAGAA |
| shFOS-2 | GCGGAGACAGACCAACTAGAA |
| shFOS-3 | GACCGAGCCCTTTGATGACTT |
