# Supplementary material for: PHF5A facilitates the development and progression of gastric cancer through SKP2-mediated stabilization of FOS
Source: J Transl Med. 2023 Jan 6;21:5. doi: 10.1186/s12967-022-03821-w (PMC9817416; doi:10.1186/s12967-022-03821-w)
Supplement: Supplementary file 2 — Additional file 2: Table S2. Primers used in qPCR. [file 12967_2022_3821_MOESM2_ESM.docx]

Table S2. Primers used in qPCR

| Gene | Forward primer sequence (5’-3’) | Reverse primer sequence (5’-3’) |
| --- | --- | --- |
| GAPDH | TGACTTCAACAGCGACACCCA | CACCCTGTTGCTGTAGCCAAA |
| PHF5A | ATCTTTTGCCGCAAGCAG | AGAGTGCAGGGACGCACATA |
| FOS | CAGACTACGAGGCGTCATCC | TCTGCGGGTGAGTGGTAGTA |
