# Supplementary material for: PHF5A facilitates the development and progression of gastric cancer through SKP2-mediated stabilization of FOS
Source: J Transl Med. 2023 Jan 6;21:5. doi: 10.1186/s12967-022-03821-w (PMC9817416; doi:10.1186/s12967-022-03821-w)
Supplement: Supplementary file 3 — Additional file 3: Table S3. Antibodies used in western blotting and Co-IP. [file 12967_2022_3821_MOESM3_ESM.docx]

Table S3. Antibodies used in western blotting and Co-IP

| Primary antibodies | Dilution in WB | Source species | Company | Catalog No. |
| --- | --- | --- | --- | --- |
| PHF5A | 12 | 1:750 | Rabbit | Abcam |
| C-FOS | 41 | 1:1000 | Mouse | Abcam |
| GAPDH | 37 | 1:30000 | Mouse | Proteintech |
| Secondary antibody | Dilution |  | Company | Catalog No. |
| HRP Goat Anti-Mouse IgG | 1:3000 |  | Beyotime | A0216 |
| HRP Goat Anti- Mouse IgG | 1:3000 |  | Beyotime | A0208 |
